# Pancreatic adenocarcinoma, chronic pancreatitis, and MODY-8 diabetes: is bile salt-dependent lipase (or carboxyl ester lipase) at the crossroads of pancreatic pathologies?

**DOI:** 10.18632/oncotarget.23619

**Published:** 2017-12-22

**Authors:** Dominique Lombardo, Françoise Silvy, Isabelle Crenon, Emmanuelle Martinez, Aurélie Collignon, Evelyne Beraud, Eric Mas

**Affiliations:** ^1^ Aix Marseille Univ, INSERM, CRO2, Centre de Recherche en Oncologie Biologique et Oncopharmacologie, Marseille, France

**Keywords:** pancreatic adenocarcinoma, chronic pancreatitis, diabetes, bile salt-dependent lipase, carboxyl ester lipase

## Abstract

Pancreatic adenocarcinomas and diabetes mellitus are responsible for the deaths of around two million people each year worldwide. Patients with chronic pancreatitis do not die directly of this disease, except where the pathology is hereditary. Much current literature supports the involvement of bile salt-dependent lipase (BSDL), also known as carboxyl ester lipase (CEL), in the pathophysiology of these pancreatic diseases. The purpose of this review is to shed light on connections between chronic pancreatitis, diabetes, and pancreatic adenocarcinomas by gaining an insight into BSDL and its variants. This enzyme is normally secreted by the exocrine pancreas, and is diverted within the intestinal lumen to participate in the hydrolysis of dietary lipids. However, BSDL is also expressed by other cells and tissues, where it participates in lipid homeostasis. Variants of BSDL resulting from germline and/or somatic mutations (nucleotide insertion/deletion or nonallelic homologous recombination) are expressed in the pancreas of patients with pancreatic pathologies such as chronic pancreatitis, MODY-8, and pancreatic adenocarcinomas. We discuss the possible link between the expression of BSDL variants and these dramatic pancreatic pathologies, putting forward the suggestion that BSDL and its variants are implicated in the cell lipid metabolism/reprogramming that leads to the dyslipidemia observed in chronic pancreatitis, MODY-8, and pancreatic adenocarcinomas. We also propose potential strategies for translation to therapeutic applications.

## INTRODUCTION

Pancreatic adenocarcinomas (PAC) and diabetes mellitus are responsible for the deaths of around two million people each year worldwide [http://www.who.int/diabetes/global-report/en/ and http://globocan.iarc.fr]. Unlike these pancreatic diseases, chronic pancreatitis is not generally a direct cause of death; patients typically die of complications such as digestive haemorrhages, except where the chronic pancreatitis is hereditary. Pancreatic cancers represent 10% of all digestive cancers; 90% of these are PAC (GloboCan 2012, http://gco.iarc.fr/today/home). The survival rate is extremely low, with a case fatality ratio of about 0.9. PAC could be the second cause of death by cancer by 2030 [1], and today has a 5-year survival rate of less than 4% in western countries [2]. Its poor prognosis is mainly due to its lack of response to currently available therapies [3, 4] and to a very low curative resection rate (15% of patients). This low curative resection rate is due to the fact that patients present with nonspecific symptoms, which, along with a lack of early biological markers, results in delayed diagnosis and metastasis formation. Risk factors for developing pancreatic cancer are multiple, and include many genetic syndromes such as Lynch syndrome and Peutz-Jeghers syndrome [5]. Chronic pancreatitis, along with long-lasting type 2 diabetes mellitus (when it is itself not a consequence of pancreatic cancer [6]), may contribute to PAC development by generating oxidative stress and DNA damage [7]. Smoking, type 2 diabetes, and chronic pancreatitis account for one-quarter to one-third of pancreatic cancers [3]. Mass sequencing studies have so far been unsuccessful in highlighting any potential genetic etiology shared between these pancreatic pathologies.

The purpose of this review is to bring together evidence connecting these pancreatic diseases, focusing on bile salt-dependent lipase (BSDL), also known as carboxyl ester lipase [CEL, 8]. BSDL is found in the pancreatic secretions of all species examined up to now, from fish to humans [9–11]. BSDL, having broad specificity, can hydrolyze triacylglycerides, esters of cholesterol, phospholipids, esters of lipid-soluble vitamins [12, 13] and ceramides [14, 15]. When it reaches the duodenal lumen, and after activation by primary bile salts, BSDL, in concert with other pancreatic lipolytic enzymes and preduodenal lipases, acts to complete the digestion of dietary lipids [12, 13, 16]. BSDL is also expressed by the lactating mammary glands of many species [17], including humans [17–21]. As an enzyme, BSDL plays an important role in milk-fat digestion and absorption in suckling newborns [22]. This role is essential, as at birth the human pancreas is unable to secrete sufficient lipolytic enzymes to ensure lipid digestion [23]. In newborns, intestinal BSDL generates lipolytic products that are toxic to trophozoites such as *Giardia lamblia*, the cause of giardiasis and severe diarrhea and malabsorption [24]. BSDL in milk also protects the intestine from fat-derived injury [25], preventing neonatal necrotizing enterocolitis [26]. The toxicity of human milk to a number of other pathogens (*Entamoeba histolytica*, *Trichomonas vaginalis*, herpes simplex virus (type II), and *Staphylococcus aureus*) has been attributed to lipids following the hydrolytic activity of BSDL [27, 28]. Although BSDL was first thought to be limited to the gastrointestinal tract, it has since been detected in the plasma of patients with diabetes [29] and normolipidemic patients [30]. BSDL (mRNA or protein) is also found in testis and adrenal glands [31], endothelial cells [32], aortic homogenates [33], eosinophils and macrophages [34, 35] and pituitary glands [36, 37]. The distribution of the enzyme throughout various tissues suggests that BSDL may have multiple effects on biological functions, particularly in cell and tissue lipid metabolism. For example, in testis and adrenal glands, BSDL may participate in the homeostasis of cholesteryl esters to provide free cholesterol for the synthesis of steroids [31]. In the pituitary gland, BSDL hydrolysis of ceramides can positively regulate pituitary hormone secretion in both normal and adenomatous pituitary cells [36]. Endothelial BSDL can hydrolyze lysophospholipids, thereby inhibiting their cytotoxic effects [32]. In human eosinophils, BSDL may prevent cells from being lysed by the large amount of lysophospholipid present in the plasma membranes of parasites [34]. Both vascular and circulating as well as BSDL originating from macrophages [33, 38, 39] may interact with lipoproteins (chylomicrons and LDL) to modulate the progression of atherosclerosis [32, 33, 35, 39]. The physiological role of the hepatic enzyme is less clear [40–42]; it may play a role in the metabolism of chylomicron remnants in the space of Disse [43]. However, the uptake of chylomicron retinyl ester by the liver and other tissues in BSDL-deficient mice generated by targeted disruption of the BSDL gene is not affected when compared to wild-type mice [44]. Bile salts particularly primary bile salts are essential to the enzyme activity on water-insoluble substrates (hence the name of the enzyme) such as cholesteryl esters [8]. However, data showed that the Platelet-Activating Factor (PAF) and (lyso)phosphatidic acid (but not (lyso)phosphatidylcholine) may also act as effective activators both on water- and lipid-soluble substrates [45]. Consequently, BSDL appears to play a role in intracellular lipid metabolism that is distinct from its action as a lipolytic digestive enzyme.

### Gene organization and BSDL structure

**Figure 1:**
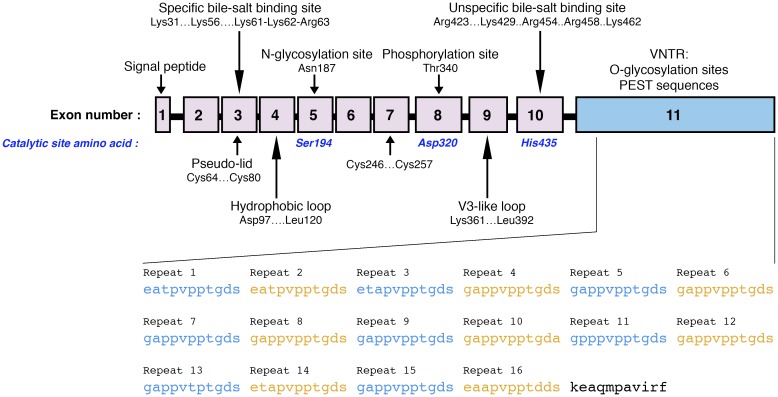
Schematic representation of the BSDL gene coding sequence Numbered boxes represent each exon of the gene. Arrows show the sequence areas that code for specific structures or sites of the protein. The amino acids involved in the catalytic site of BSDL are indicated in blue. VNTR sequences encoded by exon 11 (blue box) are displayed as full amino acid sequences.

The gene of the human BSDL is 9850 bp long. It is located at locus 9q34.3 of chromosome 9 [46], and close to the ABO locus [47]. Of the species examined to date, there is high conservation of the numbering and positioning of the intron-exon organization, and the BSDL gene consists of 11 exons [37]. Figure 1 shows the specific site or domain encoded for by each individual exon. The BSDL mRNA 5′-untranslated domain and the signal peptide are encoded by exon 1, the least conserved of the exons. Exon 2 encodes part of the heparin-binding site [11]. The two disulfide bridges present on BSDL are encoded by exons 3 and 7 [48, 49], and help to preserve the structure of the enzyme. Exons 5, 8, and 10 encode the Ser194, Asp320, and His435 residues [50–52], respectively, which constitute the catalytic site and participate in the catalysis of water-soluble and lipid-soluble substrates [53]. Facing the active site, a short loop is formed by the first disulfide bridge between Cys64 and Cys80, [53]. This bridge might act as a pseudo-lid *i.e.* a nonfunctional counterpart of the lid displayed by classical triacylglycerol lipases, which is involved in interfacial activation [55] – a process not exhibited by BSDL [56]. In addition, a basic amino acid sequence, Lys61-Lys62-Arg63, is constitutive of the specific bile salt-binding site and is encoded by exon 3 [57], while a hydrophobic domain of BSDL located between Asn98 and Leu121, which could be exposed at the surface of the protein during enzyme folding [58], is encoded by exon 4 [11]. Another loop region, generated by the consensus sequence that is between Gly117 and Glu130, is also encoded by exon 4 [11]. The binding of primary bile salt to its specific [25] (or activation [59], or proximal [54]) BSDL binding site promotes the withdrawal of this loop so that the substrate can move into the active site [54]. This explains the requirement for primary bile salts for the dimerization and activation of BSDL to occur [25]. In addition, crystal studies indicate that, when bile salt molecules are not present, BSDL has a functional oxyanion hole and a catalytic site that is preformed [60], which explains its action on water-soluble esters in the absence of bile salts [24]. Studies using recombinant BSDL, however, have suggested that dimerization might not be required for the BSDL activation by bile salts [61]. Crystal structure analysis [54], computer modeling analysis [62], and site-directed mutagenesis [63] demonstrate that modification of BSDL enzymatic activity by micellar bile salt involves the Arg63 and Arg423 residues (which do not interact with bile salts directly) via intramolecular hydrogen bonding with the C-terminal domain of BSDL. Bile salt interaction with the lid domain would disrupt bonding, causing the loop to move away [54], and therefore pulling the C-terminal domain out of the catalytic site [63]. Exon 5 encodes a *N*-glycosylation site at Asn187 [48] and exon 8 codes for the phosphorylation site at Thr340 [64]. Exon 9 codes for the V3-like loop involved in BSDL intracellular transport [65] and interaction with the CXCR4 chemokine receptor of platelets [66]. Exon 10 encodes a second cluster of basic residues from Arg423 to Lys462 characterized as the nonspecific (or distal) bile salt-binding site [67]. Exon 6 codes for structural domains that are well conserved during evolution *i.e*. helix-αD and β-sheet-6 [37].

To conclude the gene organization schema, it should be noted that the final exon, exon 11, contains a highly polymorphic GC-rich region [17]. This exon is composed of a variable number of tandem repeated identical sequences, or VNTR and codes for the C-terminal domain of BSDL [17, 68]. The C-terminal domain is formed of repeats of 11 amino acid units known as mucin-type PEST repeats (rich in proline, glutamic acid, serine, and threonine). There are variations in the number of tandem repeats between individuals, from three to 23, with 16 repeats being the most common in both Asian and Caucasian populations [68–72]. The BSDL C-terminal tail of 11 amino acid residues that follows the final repeat again shows high conservation among species [11]. Importantly, each repeat carries an *O*-glycosylated structure [48]. The physiological significance of these C-terminal repeats is still unknown. Recombinant BSDL that lacks these repeats exhibits similar activity to the native enzyme, showing that the C-terminal domain is not critical for enzymatic catalysis [70–73]. Nonetheless, the truncation by mutagenesis of the C-terminal domain suggests the involvement of this domain in bile salt modulation of the secretion [74] and catalytic activity of BSDL [63]. Moreover, bile salt activation and enzymatic activity are not dependent on *O*-glycosylation [71, 75]. However, the catalytic domain of BSDL is stabilized by glycosylation of the C-terminal repeated sequences [76]. In addition, glycosylation of this C-terminal domain may protect BSDL from duodenal proteolytic degradation [76]. Exon 11 of rat *BSDL* also codes for a sequence located before the repeats that is involved in the intracellular transport of BSDL [74]. Among species, therefore, there is high conservation of the entire amino acid sequence of the N-terminal domain [37].

In primates, there is a striking similarity between the BSDL gene and another gene [46, 77, 78]. This gene spans 4846 bp and lacks a large 5 kb segment, which, when compared to the BSDL gene, includes *BSDL* exons 2–7. In human, the tissue specificity of this transcribed gene, referred to as the *BSDL* pseudogene (*BSDLP*) [78], has been lost during organogenesis [79]. The *BSDL* and *BSDLP* genes, which are found in tandem at the same locus of chromosome 9, may be the result of gene duplication. The origins of the duplication, restricted to higher primates only [77], are unknown. It is possible that a transposon-like mechanism has occurred at some stage in the replication process [46]. This process results in an additional copy of a gene being inserted upstream from the primary location, forming a lengthy duplicated sequence at the boundary of the insertion [46]. It is probable that the original gene encoding BSDL has been inactivated during evolution and become a pseudogene [80]. The inactivation of an original gene is unusual, and in the case of BSDL it is difficult to envision what was the selection pressure involved in this process, since tissue specificity and enzyme intestinal function are well conserved among species. The transposon-like mechanism hypothesis is supported by the observation that the promoter regions of the mouse gene and human pseudogene are highly similar [46, 81]. There is no homology shared between BSDL, or its gene, and other lipases, or corresponding genes. BSDL does, however, share homology with acetylcholine esterase [17, 82], indicating the evolution of the BSDL gene as a distinct lipase/esterase gene.

### Gene regulation

*BSDL* expression is dependent on the proximal 839 bp of the 5′-flanking promoter region that encompasses the CCAAT and TATA elements [83]. Two closely located cis-elements constitute the enhancer element [84]. The proximal sub-element mediates a positive effect, whereas the distal one exerts a silencing effect on the proximal element. No homology is shared between these cis-elements and known cis-elements – the activity of the enhancer therefore appears to be dependent on currently unidentified transcription factors. Evident in the 5’-flanking region of *BSDL*, however, are consensus sequences of numerous well known transcriptional regulatory elements that respond to glucocorticoids (glucocorticoid regulatory element, GRE), sterols (sterol regulatory domain, SRD), interferon (interferon regulatory factor, IRF), and the acute-phase response element, as well as to APs and Sp1 ubiquitous transcription factor binding sites [83]. The CpG-rich islands that are frequently found in association with housekeeping gene promoter regions are not detectable in the promoter of *BSDL* [83]. Despite the restriction of pancreatic *BSDL* expression to the exocrine tissue, its regulation appears to be different from that of the elastase I gene promoter [85]. In the elastase gene, PTF-1 (pancreas specific transcription factor 1a, which plays an essential role in early and late pancreas development and differentiation) acts by binding to the A element of the gene, and restricting elastase expression to acinar cells [86]. The *BSDL* promoter contains a putative PTF-1 binding site. However, nucleotide modifications in the human promoter 5′-flanking of this PTF-1 site create a CCAAT/enhancer-binding protein (C/EBP)-like binding motif that overlaps the p64-binding site of the PTF-1 motif [87]. Such differences result in the formation of a new exocrine-specific complex in the human BSDL gene, compensating for the fact that PTF-1 is prevented from binding to the proximal site in the human *BSDL* promoter. Therefore, PTF-1, which participates in the regulation of elastase, α-amylase, and colipase expression in the pancreas [85, 86, 88], appears not to be involved in pancreatic BSDL gene activation [84, 87]. Some other putative binding sites for transcription factors along with an miRNA485-5p binding site have been identified *in silico* [37]. However, regulatory motifs on the BSDL gene still require characterization.

### Transport of BSDL in pancreatic acinar cells

The transport of BSDL in pancreatic acinar cells occurs in close association with a folding membrane complex involving Grp94 [89]. BSDL binding to glycosphingolipids in lipid rafts by means of a V3-like loop probably plays an important role in ensuring the adequate folding of the enzyme [65]. Both *N*- and *O*-glycosylation of the protein are involved in the BSDL secretion process. In order for the enzyme to become correctly folded and fully active, transfer of the N-oligosaccharide precursor on to the Asn187 residue [11, 62, 79, 81, 90–94] of the nascent BSDL is required [95]. This may be due to the fact that the catalytic site serine at position 194 and the *N*-glycosylation site at Asn187 [50] are in close proximity. Once it has been transferred as a whole to the Asn187, the *N*-glycan structure can be trimmed and matured without affecting the secretion rate of the protein [95]. The non-*N*-glycosylated BSDL fraction is directed to the ubiquitin-dependent proteasome degradation pathway [96]. Ubiquitinated BSDL (Ub-BSDL) degradation takes place at the membrane level, with no obvious retrotranslocation to the cytosol [96]. BSDL association with the membrane is triggered by ATP [97], which translocates into the endoplasmic reticulum (ER) [98] and is critical for the degradation of BSDL [96]. It therefore appears that *N*-linked glycans are critical for BSDL transfer from the ER (where transfer of the oligomannosidic structure onto the enzyme occurs) to the cis-Golgi network. Once transferred to the cis-Golgi compartment, *O*-glycosylation of the *N*-glycosylated BSDL occurs at the repeated C-terminal sequences (PEST sequences), mainly on residues of threonine [48]. Free PEST repeats signal the degradation of BSDL [99]. Thus, the enzyme that presents any free (*i.e.* non-glycosylated) PEST repeats is probably targeted for degradation. The fraction of BSDL that becomes secretion-competent upon completion of *O*-glycosylation moves to the trans-Golgi network. Association with intracellular membranes is required for transfer of BSDL to the trans-Golgi compartment from the ER [58, 100]. Protein casein kinase II [97, 101] phosphorylates the Thr340 residues of the BSDL fraction that arrives at the trans-Golgi network [64]. Thr340 phosphorylation, found in human, rat, ferret, bovine, and mouse BSDL [11], is involved in releasing BSDL from membranes [65, 97] and in allowing its movement to the secretion pathway [64, 97, 101]. Phosphorylation of the BSDL fraction that is not completely *O*-glycosylated may not occur; *in fine* it would be targeted towards the degradation pathway that involves the ubiquitin-dependent proteasome [96]. It should be noted that Thr340 phosphorylation is not essential for the activation by bile salts or enzyme activity [64]. Following its phosphorylation, BSDL is released from intracellular membranes. Later stages of the folding process involve membrane-associated Grp94 chaperone [102]; in the trans-Golgi network the chaperone probably assists in directing BSDL towards secretion [89]. The folding complex, which includes Grp94 [89], also helps to complete the *O*-glycosylation of BSDL [99] by accompanying BSDL along the secretion pathway from the ER to the Golgi [103], and by ensuring that it is kept in close contact with the membrane glycosyltransferases of the Golgi. *O*-glycosylation would not occur in BSDL molecules that Grp94 was unable to adequately accommodate and these would subsequently be targeted for degradation by the proteasome, suggesting that Grp94 could be an essential sensor in the trans-Golgi network for the correct folding of BSDL [99]. Ub-BSDL degradation could therefore either occur at the level of the trans-Golgi membranes or after a return to the ER [97]. Rab6-dependent vesicular transport may take BSDL molecules that are abnormal or incompletely *O*-glycosylated back to the ER from the Golgi [104, 105]. Therefore, two processes in pancreatic acinar cells may act as sensors of the BSDL folding stage. The first could act in the ER to separate non-*N*-glycosylated BSDL molecules. The second process could operate in the trans-Golgi compartment to sense non-*O*-glycosylated BSDL molecules. Both processes would therefore direct the abnormal BSDL molecules to the ubiquitin-dependent proteasome degradation pathway.

Essentially, phosphorylation at Thr340 [64] frees BSDL from intracellular membranes, sorting the phosphorylated secretion-competent BSDL molecules from the non-phosphorylated ones. These latter molecules are consequently secretion-incompetent and are potentially returned to the ER for more cycles of *O*-glycosylation, or targeted for degradation by the proteasome. Grp94, which senses the correct folding of BSDL, could be involved at this level [89]. BSDL molecules sensed as being secretion-competent are subsequently directed to the condensing vacuoles and zymogen granules of the secretory compartment. *O*-glycosylation seems to be important not only for intracellular transport/secretion and for masking PEST sequences [8], but also for the solubility of the protein, by preventing self-association due to hydrophobic areas exposed at the surface of the enzyme [61].

Importantly, the glycosylation of BSDL differs depending on the pathophysiological status of the pancreatic acinar cells [106, 107]. The fate of the enzyme in pathophysiological conditions that might affect its transportation in pancreatic acinar cells should therefore be examined.

### BSDL and pancreatic pathologies

### Diabetes

Diabetes is a chronic disease that occurs when the endocrine cells of the pancreas do not produce enough insulin, or when the organism is unable to properly use insulin to regulate blood sugar concentration. In 2012, diabetes mellitus was the direct cause of 1.5 million deaths [http://www.who.int/diabetes/global-report/en/].

Autoantibodies specifically directed towards BSDL are found in patients with diabetes. This indicates that there are certain situations in which the enzyme, or the degradation products of the enzyme, is identified as nonself [108]. The prevalence of these autoantibodies against BSDL demonstrates that the exocrine tissue of the pancreas might be affected in type 1 autoimmune diabetes at onset, and in first-degree relatives of diabetic subjects [108]. The origin of such antibodies is puzzling. The high blood glucose concentrations found in patients with diabetes and during the postprandial phase in people with prediabetes may affect the metabolism of sugars and impact the hexosamine biosynthesis pathway. The metabolism of acinar cells may also be affected by blood glucose level, as seen in human hepatoma cells [109], resulting in glycosylated entities that are immunogenic. The modification of BSDL glycosylation, in particular the *O*-glycosylation of PEST repeats, influences its intracellular transport and degradation [71, 75, 99], and may result in antigenic glycopeptides generated by protein degradation being exposed at the surface of acinar cells and presented to the immune system*.* Considering that antibodies are not generated against circulating BSDL [108, 110, 111], the membrane presentation of these antigenic glycopeptides could be essential. Both modifications of the sugar metabolism of acinar cells and membrane presentation of glycopeptides may explain the presence of autoantibodies directed against the C-terminal domain of BSDL in some patients with type 2 diabetes [108]. However, the presence of autoantibodies against BSDL in first-degree relatives without clinical diagnosis suggests the involvement of genetic factors [108].

**Figure 2:**
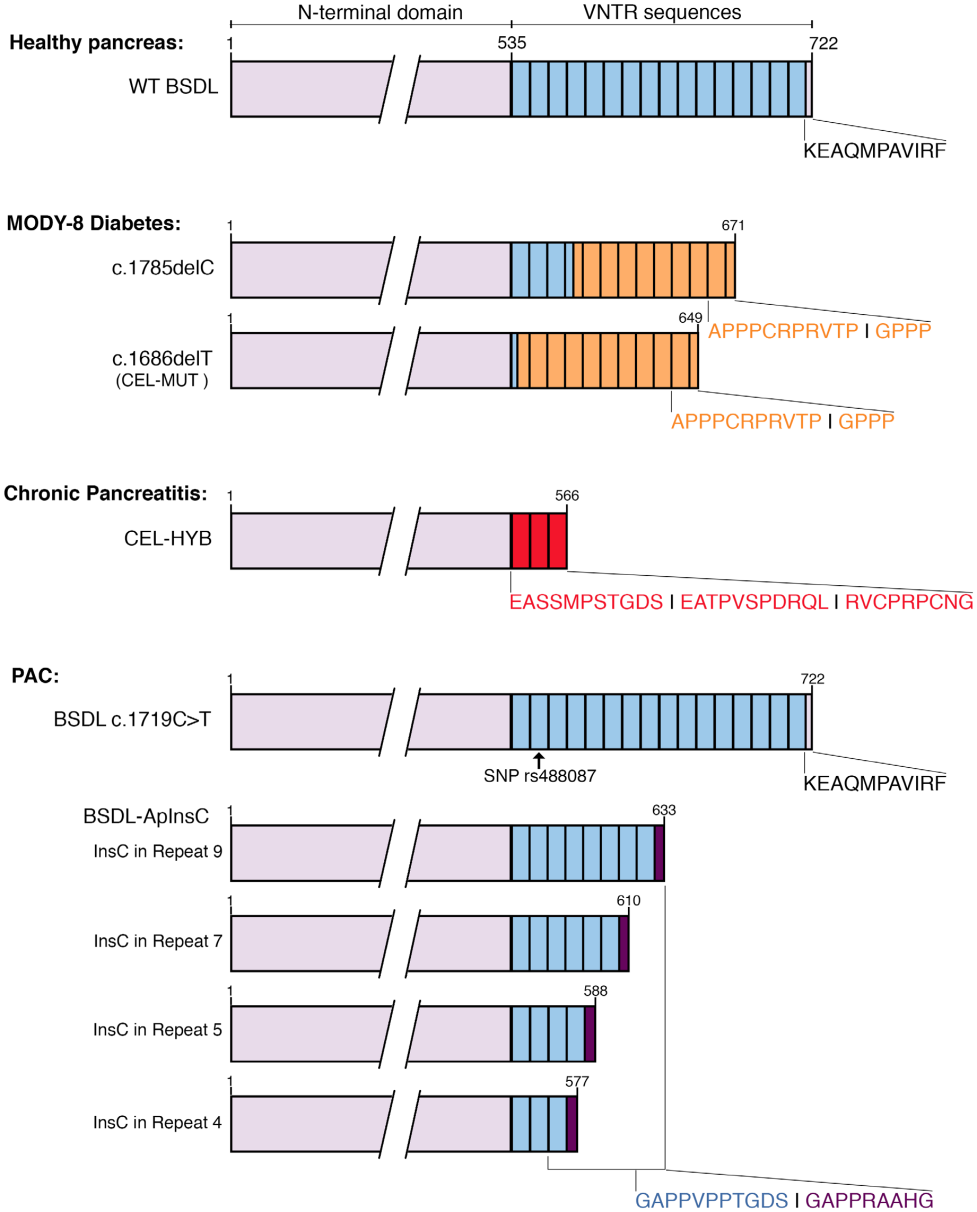
Schematic representations of human BSDL variants and associated pathologies The sequences of exons 1 to 10 encoding the N-terminal domain of BSDL are represented in purple. VNTR sequences are in blue; each box represents a single VNTR. The sequences of the VNTR that are modified upon mutation (i.e., insertion, deletion), or recombination are colored in orange (MODY-8 diabetes), red (chronic pancreatitis), or dark purple (PAC), along with the corresponding C-terminal sequence of the protein.

Circulating antibodies to BSDL are also detected in an autosomal dominant inherited syndrome of exocrine dysfunction and diabetes (personal observation). This syndrome, now referred to as MODY-8 (maturity-onset diabetes of the young, type 8), is due to a single-base deletion mutation in the *BSDL* VNTR (c.1686delT or c.1785delC) (Figure 2) detected in two independent Norwegian families [112]. This syndrome affects both acinar and beta pancreatic cells [112]. Fat deposition (lipomatosis) is observed in the pancreas of the mutation carriers before the disease is diagnosed [113]. This pathogenic process, associated with the expression of mutated BSDL and an upregulation of mitogen-activated protein kinase signaling [114], takes place in the exocrine pancreas early in infancy, ultimately leading to diabetes when people reach their forties. The c.1686delT mutation appearing in the first VNTR of the *BSDL* leads to a frameshift, and consequently to modification of the C-terminal domain of the protein (referred to as CEL-MUT) [112]. Norwegian studies further show that CEL-MUT and wild-type BSDL display post-translational modifications and similar rates of secretion. However, CEL-MUT is misfolded and is prone to form aggregates leading to activation of the unfolded protein response (UPR) in a HEK293 cell model transfected with the cDNA encoding CEL-MUT [115, 116]. Once secreted, CEL-MUT lines the outside plasma membranes. The aggregated protein undergoes a robust reuptake, which occurs after secretion. This reuptake, by both exocrine and endocrine cells, is followed by transport to the lysosome where the protein is degraded. Furthermore, the viability of both acinar and beta cells is affected upon capture of aggregates [117]. This leads a mixed distribution of the protein in cells, with mutated BSDL present in secretory pathway vesicles (BSDL associated with Grp94) and in vesicles of the endocytic pathway (BSDL in early endosomes and lysosomes) [117]. Torsvik *et al.* [117] reported that CEL-MUT was not degraded via the autophagosomal pathway. The absence of autophagy suggests that aggregates do not form during CEL-MUT synthesis but rather after its release outside the acinar cell. Once reuptaken, the protein is transported to the lysosome degrading compartment [117]. Consequently, CEL-MUT affects the viability of both acinar and beta cells, providing a possible (albeit simplistic) explanation of the specific symptoms of this pathology [116, 117]. Studies show that CEL-MUT forms intracellular aggregates, increases ER stress, and induces UPR activation in rat pancreatic AR4-2J cells [116]. The UPR then induces NF-*k*B activation and promotes apoptosis [116].

In any case, if CEL-MUT variant aggregates are poisonous for cells – and particularly for beta cells in the pancreatic gland – it is assumed [115, 117] that these aggregates might be formed at the basolateral region of the membrane in order to be taken up by proximal beta cells.

Pancreatic acinar cells are polarized cells, which enables the flow of pancreatic enzymes within pancreatic ducts. If impairment of protein transport or modification of secretory pathways occurs in CEL-MUT-expressing pancreatic acinar cells, this may explain the retention of the truncated protein within the cell and the formation of aggregates (further presented at the cell surface). In physiological conditions, BSDL present in the duodenum after pancreatic secretion crosses the intestinal barrier by means of a transcytosis mechanism [118, 119] to reach the bloodstream [29, 30]. The protein is then cleared from the blood by renal glomerulus filtration, and is detected in the urine of healthy subjects [120]. Only some variants of mutated BSDL are detected in patients’ urine [115], with the exception of CEL-MUT and CEL-Ins4 [115], likely due to antibodies used in this study and/or to the low rate of CEL-MUT secretion [112]. Interestingly, Xiao et al. [116] have suggested that the intracellular accumulation of CEL-MUT in acinar cells can lead to chronic pancreatitis, the earliest symptom observed in childhood [113, 114], ending with MODY-8 diabetes [69, 112].

It is therefore proposed that MODY-8 is defined as a protein misfolding disease resulting from the expression of mutated BSDL [115, 116]. However, results of Xiao et al. [116] could be partially overstated, as the commercial cDNA encoding CEL and CEL-MUT used to transfect cell models in this study contains a three base-pair in-frame deletion (p.E365del). Although E365 is well conserved during evolution, the deletion of this amino-acid residue located in the surface loop consisting of αI and αJ helixes [37] seems not to affect the enzyme activity, and is not sufficient by itself to trigger activation of the UPR and cell death [121]. *In fine*, CEL-MUT causes chronic pancreatitis, meaning that the MODY-8 diabetes symptoms are not isolated effects of CEL-MUT expression [116]. Nonetheless BSDL knock-out mice do not develop pancreatic dysfunction [122]*.* In light of the proposed mechanism leading to the pathology with the retention of the misfolded protein and the activation the UPR answer [115], the absence of the CEL-MUT expression therefore explains this negative result. Transgenic mice expressing CEL-MUT also do not develop pancreatic dysfunction [123]. Many reasons may explain this negative result: 1) the mouse models may not be relevant for this particular pathology, as its development takes place over many years; 2) the genetic background may obscure the effects of mutation [123]; and finally, 3) the role of BSDL may vary between species, possibly due to differences in the number of repeats [11].

### Pancreatitis

In this review, we will not discuss acute pancreatitis, in which the serum level of many pancreatic enzymes is greatly increased. Indeed, serum levels of BSDL are significantly increased in patients suffering from acute interstitial pancreatitis or necrotizing pancreatitis [110]. A high serum level of BSDL, therefore, even where serum levels of amylase are normal [124], suggests acute pancreatitis [125].

Progressing over time, chronic pancreatitis is a pancreatic inflammatory disease. Clinicians have no therapeutic solution to prevent recurrent episodes and progression to chronic pancreatitis. This absence of effective therapy could in part be due to limited knowledge of the pathophysiology of this disease. People with genetically susceptible backgrounds are prone to develop chronic pancreatitis once exposed to environmental factors (smoking, alcohol) [126]. The risk of pancreatitis increases with alcohol consumption [127], which disrupts defense mechanisms and/or enhances injury-associated pathways through alteration of gene expression*.* BSDL, which displays fatty acid ethyl ester- and cholesteryl ester generating activity [128–130], may be involved in this process [131]. Indeed, normally found in zymogen granules within the acinar cells of the pancreas, BSDL is also detected in the cytoplasm during acinar cell necrosis [132]. Alcohol, by increasing the expression of lysosomal and digestive pancreatic enzymes, also increases organelle fragility, which could result in prematurely activated digestive enzymes that can damage the tissue integrity.

Most reported genes associated with chronic pancreatitis that have been identified up to now code for proteins linked to the protease-antiprotease system of acinar cells, such as the cationic and anionic trypsinogen (PRSS1 and 2), chymotrypsinogen C (CTRC), carboxypeptidase A1 (CPA1), or the Kasal-type 1 protease inhibitor (SPINK1) [133]. None of these genes are associated with pancreatic adenocarcinoma, at least in German patients [134]. Albeit associated with modest odds ratios, the ABO, FUT2, and CFTR loci represent risk factors for chronic pancreatitis [133, 135].

Although VNTR numbers in the BSDL gene do not represent a risk factor for alcoholic and idiopathic chronic pancreatitis [136, 137], a recombined allele resulting from nonallelic homologous recombination occurring between BSDL intron 10 and intron 10’ of its tandemly linked BSDL pseudogene, *BSDLP,* has to be added to the list of genes involved in nonalcoholic chronic pancreatitis [138]. This recombined allele, referred to as *CEL-HYB* (Figure 2), was first discovered in a cohort of patients of European ancestry with familial chronic pancreatitis, and further detected in patients with idiopathic chronic pancreatitis [138]. *CEL-HYB* was present in 0.7% of the control cohorts and in 3.7% of the case group cohort, underlining a substantial chronic pancreatitis risk associated with the recombined allele (odds ratio = 5.2). Among reported CEL-HYB-positive subjects, none expressed the mutated *PRSS1* risk variant [138]. The *CEL-HYB* allele encodes a chimeric BSDL protein; the protein is 589 amino acids long, with a functional catalytic site and a truncated C-terminal domain with three repeats encoded by a short VNTR originating from the BSDL pseudogene with a modified C-terminal amino acid sequence (*i.e.* RVCPRPCNG instead of KEAQMPAVIRF [11]). This chimeric protein shows reduced cellular clearance and decreased secretion rate [138] suggesting that the chimeric protein may be more stable than the wildtype protein. An alternative hybrid allele, which codes for another chimeric CEL (*CEL-HYB2*), has been characterized in three independent cohorts from China, Japan, and India [139]. However, this variant, which harbors premature stop codon within its chimeric exon 10, is devoid of VNTR [142]. *CEL-HYB2* is not associated with chronic pancreatitis. *CEL-HYB* [138] (also referred to as *CEL-HYB1* [139]) is not detected in Asian populations [139] and could be a chronic pancreatitis risk factor specific for Caucasian populations.

In fact, CEL-HYB shows impaired secretion, decreased activity on synthetic substrates, and retention within acinar cells. The observed increase in the LC3-II autophagy marker in human cell models shows that the retention of the chimeric protein promotes autophagy [138], a dysfunction described as a key event in pancreatitis [140, 141]. Autophagy associated with chronic pancreatitis cooperates with Kras to promote pancreatic cancer [142]. Most carriers of CEL-HYB in the population do not develop pathology, suggesting that other factors may participate in the inflammatory process and in the susceptibility of individuals [143, 144]. On the other hand, Xiao *et al.* [116] showed that CEL-MUT forms insoluble aggregates, likely due to the formation of inappropriate disulfide bridges. These aggregates are the cause of ER stress and trigger the UPR in the acinar cell model [116], promote elF2α phosphorylation [115], induce cell death by apoptosis, and promote NF-*k*B activation [116]. ER stress and NF-*k*B activation are early pathogenic features in pancreatitis [145]. As suggested above, pancreatitis could be an early event in MODY-8 diabetes. It should be noted that no case of diabetes is described in subjects expressing CEL-HYB [138].

### Pancreatic adenocarcinoma

The most frequently occurring pancreatic cancers are PAC, and arise from preneoplastic lesions of the pancreas called pancreatic intraepithelial neoplasia (PanIN). Larger lesions, such as intraductal papillary mucinous neoplasms (IPMN) and mucinous cystic neoplasms, may also be PAC precursors. PAC are complex diseases involving genomic, epigenetic, and metabolic modifications. They exhibit aberrant signaling pathways promoting cell survival, proliferation, migration, and invasion, ending with the formation of distal metastases.

A genome-wide association study (GWAS) meta-analysis identifies several susceptibility loci in the p21 region of chromosome 9 that are associated with the pathogenesis of multiple cancers, including pancreatic cancer [146]. Single nucleotide polymorphisms (SNPs) at this locus may have heterogeneous effects *i.e.* result in increased or decreased susceptibility. Albeit modestly, the risk for pancreatic cancer is also associated with another locus on chromosome 9, the ABO locus in the q34 region, which was the first common pancreatic cancer risk locus [147] and controversially associated with patient survival [148–152] Although the cohort size was too small to reach genome-wide significance, data of Qi *et al.* [153] strongly suggest that the ABO locus also presents linkage with type 2 diabetes. Yet GWAS susceptibility variants are unlikely to explain the potential genetic etiology shared between type 2 diabetes and pancreatic cancer [154]. To the best of our knowledge, only one publication links chronic pancreatitis to ABO blood type-B groups in Northeastern German population. In this population, group O appears to have protective effects [155]. Germline or somatic mutations that are present in the GC-rich segment of chromosome 9 where the BSDL gene is located are not accessible by mass sequencing [156], and careful examinations of electropherograms obtained after Sanger sequencing are essential to detect them.

### Germline and somatic mutations

Pancreatic cancers are characterized by many somatic mutations, some of which are particularly prevalent [3]. The most common is that of the Kras oncogene which appears at the beginning of the carcinogenesis. It takes more than ten years from the appearance of the first mutation for the pathology to be established. We recently reported that a c.1719C > T transition (SNP, *rs488087*) can be detected in *BSDL* VNTR (Figure 2) [157]. This SNP could be a useful marker for defining a population at risk of developing pancreatic cancer (occurrence: 63.90% in the pancreatic cancer *versus* 27.30% in the control group) particularly if associated with other potential risk factor of PAC [158]. This SNP needs to be validated in a larger cohort of patients and healthy people before being used as a tool to predict pancreatic cancer [158]. The odds ratio of 4.7 for the T allele is larger than those determined for other SNPs thought to be predictive of pancreatic cancer [158]. The transition c.1719C > T occuring in the third position of a codon, results in no change of encoded amino acid residue (synonymous SNP). The protein sequence is thus unchanged. Our *in silico* studies [158] indicate that the SNP rs488087: 1) does not seem to induce alteration of mRNA splicing; and 2) may induce a change in the secondary structure of the BSDL mRNA, and thus has a possible impact on the processing of RNA and translation into protein. The transition c.1719C > T also generates a loss of binding site for the miRNA miR-564, which has been further shown to have no impact on BSDL expression. Examining pancreatic tissue of patients afflicted with a PAC suggested that the T allele may correlate with Kras G12R/G12D somatic mutations. These Kras mutations are associated with a bad prognosis and reduced survival [159–161].

Although VNTR length polymorphisms do not represent a risk for pancreatic cancer [162], a BSDL transcript in which a cytosine residue is inserted into the VNTR (BSDL-InsC) was recently detected in a cohort of French patients with PAC [163]. This insertion, which could not be detected in DNA extracted from blood samples from a cohort of control individuals [163], gives rise to a premature stop codon, resulting in a truncated protein and a modification of the C-terminal amino acid sequence – that is, GAPPRAAHG instead of KEAQMPAVIRF (Figure 2) [11, 163]. While all human tissue samples are positive to anti-PAVIRF antibodies, in this study 72.2% of pancreatic tumor tissue samples reacted positively with anti-PRAAHG antibodies, particularly in dysplastic areas of the tumor in contrast to neoplastic cells with ductal differentiation. Examining a cohort of normal Norwegian individuals, Raeder et al. [112] detected a germline insertion of one base in certain VNTR of BSDL with an allelic frequency of some 10%. Although the outcome of these individuals is unknown one hypothesis to explain this allelic frequency in the Norwegian population could be a genetic variation between populations of Northern [112] and from Southern [164] Europe. This last point may be illustrated by many studies in part those of Tian et al. [164] and Seldin et al. [165]. Around 70% of PanIN tissue samples were also reactive to anti-PRAAHG antibodies, suggesting that the C insertion occurs early during pancreatic carcinogenesis. Anti-PRAAHG antibodies are exclusively reactive to a truncated variant of BSDL. The truncated BSDL reactive to antibodies raised against the PRAAHG sequence (BSDL-InsC) was present in the pancreatic juice of patients with PAC [165]. Yet the low secretion rate that is due to the retention of truncated BSDL, as observed in SOJ-6 tumor cell model, partly results from an impaired transport [104]. Nonetheless, detection of truncated BSDL reactive to antibodies against the PRAAHG C-terminal sequence in pancreatic juice or in pancreatic biopsies may be a new tool in the early diagnosis of PAC.

### *O*-glycosylation alterations of BSDL

The fetoacinar pancreatic protein or FAPP, characterized by a glycotope recognized by J28 monoclonal antibodies and thus designated the J28 glycotope, is a specific component of acinar cells of the human pancreas associated with ontogenesis and development of the gland [166]. Maximum synthesis of FAPP [167] occurs when acinar cell proliferation is maximal, then declines until parturition [168]. Interestingly, FAPP also appears during carcinogenesis [168, 169]. FAPP presents many homologies with BSDL [172] and its cloning from human pancreatic tumor cells [170] indicates that the sequence of the N-terminal domain of FAPP is identical to that encoded by exons 1-10 of *BSDL*. The sequence that corresponds to exon 11 (which itself encodes the BSDL tandem repeats), however, is deleted by 330 bp and codes for six repeats. FAPP exhibits decreased activity both on synthetic substrates and on cholesteryl ester [169]. Therefore, FAPP has been characterized as a genotypic and phenotypic variant of BSDL bearing the J28 oncofetal glycotope [171]. FAPP is now referred to as pathological BSDL-J28^+^ (pBSDL-J28^+^) [172, 173]. pBSDL-J28^+^, which is *N*- and *O*-glycosylated [170] and phosphorylated [64], is poorly secreted by pancreatic tumor cells [96, 104, 168, 174]. In tumor cells, pBSDL-J28+, which is typically membrane-associated, distributes within the plasma membrane and the ER and Golgi membranes [174]. This protein retention, which occurs alongside modifications in the expression of glycosyltransferase resulting from neoplastic processes in the pancreas [175], could result in J28 glycotope formation; this requires the α(1-3/4) fucosyltransferase and the Core 2 β-1, 6-N-acetylglucosaminyltransferase [176]. These enzymes are typically found in the trans- or cis-Golgi compartments [177]. As previously observed in PAC, MODY-8 diabetes, and chronic pancreatitis, aberrant variants of BSDL (BSDL-InsC, CEL-MUT, CEL-HYB) with impaired secretion should either be degraded [96] or presented at the cell surface as aggregates and further degraded after recapture [117]. However, it is not known whether these aberrant variants of BSDL are likely to bear the J28 epitope. How such expression and cell retention of BSDL variants end up with pancreatic dysfunction and diseases is an open question (see diabetes section above).

### Immunogenicity of pBSDL-J28^+^ and potential applications

The pBSDL-J28^+^ captured by human immature dendritic cells (iDC) *via* the mannose receptor is degraded in late endosomes [172, 173]. Glycopeptides derived from this degradation, including the one bearing the J28 glycotope, may then be expressed and presented by MHC II for presentation to naive T cells. Human and murine DC loaded with pBSDL-J28^+^ or with the C-terminal domain of BSDL bearing the J28 glycotope (C-ter-J28^+^) induced the proliferation of CD4^+^ and CD8^+^ T-lymphocytes [172, 173, 178]. In addition, we showed in the murine model that this proliferation accompanies IFN-γ expression and expansion of granzyme B-expressing CD8^+^ T-cells [178]. These findings demonstrate that interactions of pBSDL-J28^+^, expressed on tumor pancreatic tissue, with DC may lead to adequate antigen trafficking and processing, and result in T cell activation. This makes it a candidate for antitumor DC-vaccination. Indeed, prophylactic and therapeutic C-ter-J28^+^-DC–vaccinations reduce ectopic tumor growth induced by the syngeneic tumor pancreatic line, Panc02 cells, provide long-lasting protection from Panc02-tumor development in 100% of C57BL/6Jr mice, and attenuate orthotopic progression of tumors and metastases as revealed by magnetic resonance imaging [178]. C-ter-J28^+^-DC-vaccinations represent a potential adjuvant therapy for patients with PAC.

### Biological effects of BSDL variants bearing the 16D10 glycotope and potential applications

In addition to the J28 glycotope, the BSDL variants may carry another tumor-associated carbohydrate antigen (TACA). This glycosylated epitope recognized by 16D10 monoclonal antibodies (mAb16D10) is thus designated the 16D10 glycotope [179–181]. The 16D10 glycotope is linked to the blood group A antigen [182]. The J28 and 16D10 glycotopes are formed due to cell sugar metabolism modifications that occur during carcinogenesis. While undetectable at the plasma membranes of normal cells, 16D10 TACA can be presented at the surface of pancreatic tumor cells (as can the J28 glycotope) [179]. How this reactive material is presented at the cell surface remains to be determined. There are a number of non-exclusive possibilities, including membrane presentation [183] of the glycopeptides that are generated by degradation of the BSDL variants could be facilitated by Grp94, which assists BSDL in folding and in intracellular transport [8]. Another possibility is the generation of putative sites for palmitoylation and membrane anchoring in repeat sequences, as detected *in silico*, of some variants of BSDL. A third possibility is that aggregated material is presented, as described for CEL-MUT [115, 116]. Analysis of the biological effects of mAb16D10 reveals interesting information [181]. The mAb16D10 decreases pancreatic tumor growth *in vivo* by triggering antibody-dependent cell-mediated cytotoxicity (ADCC). In addition, mAb16D10 triggers a cell-cycle arrest in the G1/S phase, by modifying the expression of cyclin D1, p53, or GSK-3β, followed by cell death. Anchoring of the 16D10-bearing-BSDL variant by means of fatty acid in lipid rafts acting as a signaling platform [184] may explain the apoptotic effects of mAb16D10 [181]. Such antibodies could serve to detect TACA in addition to their potential use as therapy to fight tumor cells.

### Role of vascular BSDL and variants

In physiological conditions BSDL present in the duodenum after pancreatic secretion crosses the intestinal barrier [118, 119] to reach the bloodstream [29, 30]. Vascular BSDL binds to the CXCR4 platelet receptor *via* its V3-like loop [66] and acts as a weak agonist pseudo-chemokine to optimize the aggregation of platelets and the formation of venous thrombosis [65, 185]. This favors the formation of pre-metastatic niches [186]. In the context of neoplastic diseases, in particular pancreatic cancers, it is reasonable to assume that BSDL, which forms an equimolar complex with ApoB100 in low density lipoprotein (LDL) [30], can be captured by cancer cells *via* the over-expressed LDL receptor [187]. Also, BSDL present in microparticles [185] and/or exosomes [188] released by pancreatic cancer cells can be captured *via* endocytosis or by means of fusion with the plasma membrane [188]. This holds true for noncancer cells residing in the tumor, such as fibroblasts or immune cells [189]. The presence of BSDL in microparticles [185] and exosomes [188] could exacerbate the thrombotic events that are frequently observed in patients with PAC [190]. Thrombosis may be essential for cancer cell dissemination and metastasis formation [191]. Thus, once established, the tumor may benefit from circulating BSDL.

### Impact of BSDL variants activity on lipid metabolism

### Role of vascular BSDL in lipid metabolism

Several lines of evidence point to the impact of vascular BSDL on lipid metabolism. Vascular BSDL modulates lipids associated with LDL and high-density lipoprotein (HDL), resulting in cellular protection against the cytotoxic effects of lysophosphatidylcholine, and plays a role in the induction of proliferation and cell migration [39, 192]. Moreover, as BSDL can convert large LDL to smaller, more atherogenic LDL entities, it may play a role in atherogenesis [193]. An association between the number of C-terminal repeats and serum cholesterol profile is reported, and individuals carrying a short allele of BSDL have lower total cholesterol and LDL cholesterol levels than subjects carrying two common alleles [194], meaning that BSDL variants may have implications in vascular lipid metabolism. The vascular role of BSDL is strengthened by the observation that BSDL can be activated by physiological concentrations of platelet-activating factor [45]. The ABO blood group locus, which is proximal to the BSDL locus, is also associated with lipid phenotype [195–197]. Thus, it is hypothesized that the association between lipid profile and the ABO locus could be due to BSDL gene polymorphism [46].

### Role of cell BSDL in lipid metabolism

Lipids are essential cell components that provide cells with energy, act as signaling intermediates, and build biological membranes. Lipids are permanently recycled and redistributed within cells where they orchestrate apoptosis [198], autophagy [199], and inflammation [200]. A pancreatic cell model chronically treated with ethanol showed a specific accumulation of BSDL in the cytosol compartment [201], where BSDL may affect cholesterol metabolism leading to cholesteryl ester storage within cytosolic lipid droplets [202]. A similar role in cholesterol homeostasis is attributed to BSDL expressed in steroidogenic tissue, adrenal glands and testis [31], and macrophages [35]. All BSDL variants characterized to date are partly retained within acinar cells and present with a decreased activity (generally determined on synthetic substrates), but specificity studies are rare [203].

### Lipid metabolism modifications in pancreatic pathologies

Fat infiltration is an early event in nondiabetic MODY-8 VNTR mutation carriers with signs of exocrine dysfunction [114] that meet the criteria for chronic pancreatitis [116]. Dyslipidemia is associated with an inflammatory status in patients with diabetes [204]. Truncated BSDL activity may be affected as the C-terminal domain modulates its activity [63, 74]. It is known that branched fatty esters of hydroxyl fatty acids (FAHFAs) present protective effects against diabetes, with anti-inflammatory activities [205]. A recent study demonstrates that FAHFAs are the preferred substrate for CEL-MUT [203]. Any perturbation in the BSDL variant homeostasis, associated with different enzymatic properties, leading to its retention within the cell, could induce active degradation of these protective endogenous FAHFAs. Suppression of the anti-inflammatory activity of FAHFAs upon BSDL hydrolysis may cause diabetes. Overall, these data suggested that deletion in VNTR, such as c.1686delT, leads to modification of the CEL specificity. Therefore, it can be hypothesized that any BSDL/CEL variant with VNTR mutations may have different specificity than that of the non-mutated enzyme.

Fatty pancreatic infiltration is a risk factor for early lesions in pancreatic cancer [206], and lipid metabolism is dysregulated in PAC [209–211]. Metabolic reprogramming occurs in tumor cells to foster their rapid proliferation, their survival, and the formation of metastasis [207]. This reprogramming also supports the inflammation observed in neoplastic pathologies [208, 209]. Fatty acid synthase (FASN, also known as onco-antigen 519), a key lipogenic enzyme involved in *de novo* lipid biosynthesis, is significantly upregulated in pancreatic tumor cells, which results in a significantly poor prognosis of patients [210]. It is now clear that patients with pancreatic cancers present markers of tissue wasting [211] at a stage when the tumor is not yet clinically detectable, and therefore these markers present potential interest.

While the expression of BSDL – and likely that of any variant – is decreased in differentiated PAC [182], BSDL variants could play an important role in pre-neoplasia [163]. However, variation in BSDL expression levels may not be so central to pancreatic pathologies such as chronic pancreatitis, MODY-8, and PAC. Most relevant is which BSDL variant(s) is (are) expressed, and what its (their) activity and specificity is on relevant physiological substrates.

**Figure 3:**
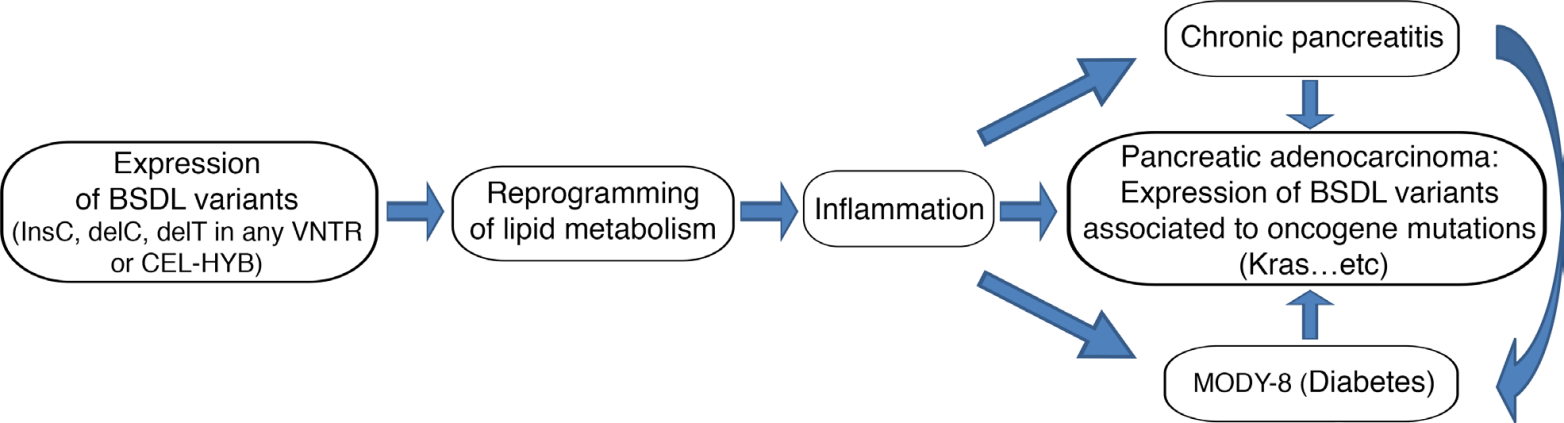
Schematic representation of the putative link between pancreatic pathologies and the variants of BSDL This shows the potential pathophysiological consequences of expression of human BSDL variants.

Collectively, all these data suggest that BSDL variants (BSDL-InsC, CEL-MUT, CEL-HYB, BSDL-J28^+^, and any other not yet characterized variant that cannot be detected by mass sequencing or “omics” studies) that are expressed and retained within acinar cells, or present in blood circulation and captured by any cell within the tumor, could be implicated in the cellular lipid metabolism reprogramming observed in pancreatic pathologies [204, 206–208]. For example, BSDL associated with LDL can be captured by pancreatic cancer cells by means of the over-expressed LDL receptor and can therefore participate in lipid metabolism reprogramming. A strong link between chronic pancreatitis, diabetes, and PAC may therefore reside in lipid metabolism modifications. These modifications can result in exocrine cell dysfunction and inflammatory processes. This may further induce chronic pancreatitis and/or MODY-8 diabetes, with pancreatic cancer then most likely to occur when other predisposing factors exist (perhaps SNP *rs488087* on *BSDL*?) in conjunction with mutations such as those of Kras (Figure 3).

## CONCLUSIONS

**Figure 4:**
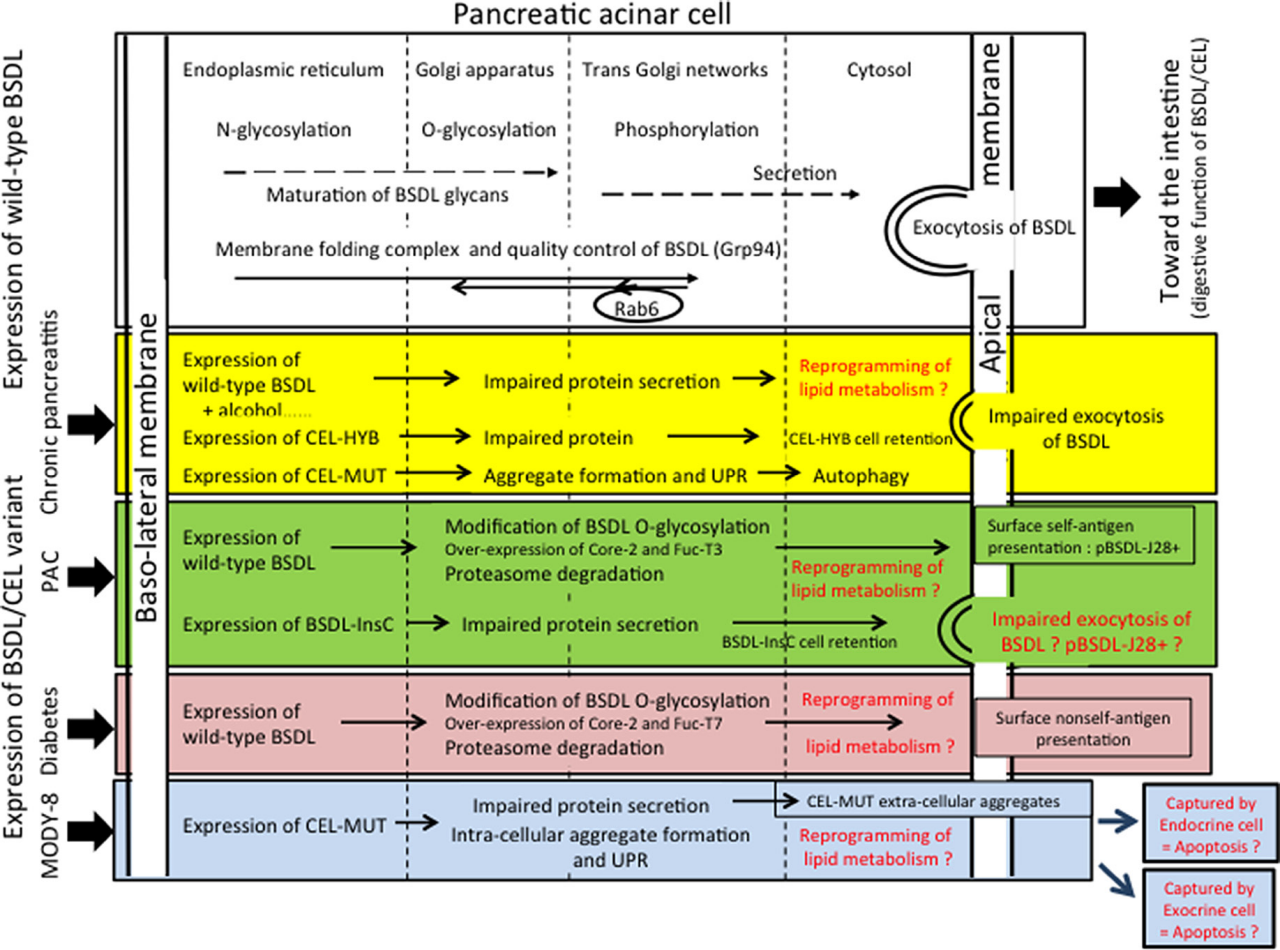
Tentative model to illustrate the involvement of the expression of BSDL/CEL variants and that of wild-type BSDL/CEL bearing post-translational modifications in pancreatic diseases Note that post-translational modifications of BSDL variants may also be affected by the pathophysiological status of exocrine cell. In red characters are putative mechanisms impacting the pathophysiological status of exocrine or endocrine pancreatic cells. Core 2, β (1-6) *N*-acetylglucosaminyltransferase; Fuc-T3 and Fuc-T7, Fucosyltransferase 3 and 7; UPR, unfolded protein response.

The expression of variants of BSDL appears to be a convergent point for chronic pancreatitis, diabetes, and pancreatic adenocarcinoma (Figure 4).

The findings obtained up to now show that: 1) a deletion of a single base within the *BSDL (CEL)* gene segment encoding VNTR leads to exocrine dysfunction and MODY-8 symptoms with beta-cell failure and pancreatic exocrine disease [112], 2) an insertion of a cytosine within any VNTR of *BSDL* leads to the expression of a new BSDL isoform in dysplastic areas and pre-neoplastic lesions of the exocrine pancreas [163], 3) a recombined allele of the *BSDL* gene and *BSDL* pseudogene confers susceptibility to chronic pancreatitis [138], 4) all these mutations or recombinations in *BSDL* are predicted to give rise to DNA encoding truncated proteins with a functional catalytic site.

Therefore, variants of BSDL/CEL with different C-terminal domains may be expressed as active monomers that have different activity/specificity than the wild-type BSDL, as seen with CEL-MUT [203]. These variants, that are partly retained within cells, may impact differently on the cell lipid metabolism involved in the development of pancreatic pathologies. This hypothesis, which opens new paths for research, is compatible with a progression of pancreatic disease development that is: expression of a misfolded BSDL/CEL, impaired secretion and degradation of the protein, leading to apoptosis/autophagy [115, 116, 138], or to reduced viability of acinar cells [117].

Such sequential mechanisms can lead to pancreatic pathologies in which active BSDL/CEL variants present within the acinar cells may play a central role together with an impaired protein homeostasis impacting the cell/tissue lipid metabolism (such as hydrolysis of FAHFAs, cholesteryl ester synthesis, etc), which *in fine* results in lipid metabolism reprogramming and in inflammatory processes followed by cell failure, cell death, or carcinogenesis.

Therefore, targeting BSDL activity by means of inhibition might be an option in developing new therapies against these pancreatic pathologies. From this perspective, the use of BSDL-specific inhibitors such as carbamates, WWL92 [212], and N-butyl-N-methyl-4-nitrophenyl carbamate [213], might be explored as potentially useful drugs.

## Abbreviations

BSDL: bile salt-dependent lipase
CEL: carboxyl ester lipase
FAPP: fetoacinar pancreatic protein
PAC: pancreatic adenocarcinoma
MODY: maturity onset diabetes of the young
SNP: single nucleotide polymorphism
